# Supplementary material for: Serum Interleukin-8 in Patients with Different Origin of Intra-Abdominal Infections in Perioperative Period
Source: Med Sci (Basel). 2019 Sep 8;7(9):94. doi: 10.3390/medsci7090094 (PMC6780305; doi:10.3390/medsci7090094)
Supplement: Supplementary file 1 [file medsci-07-00094-s001.pdf]

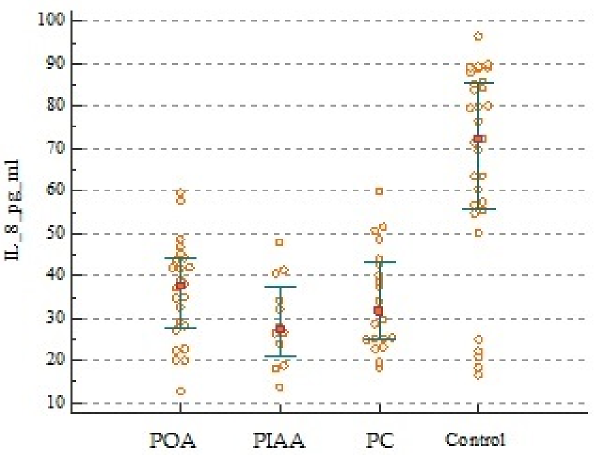

Figure S1. Distribution of the serum IL-8 level in patients with different origin of IAI before surgical intervention.

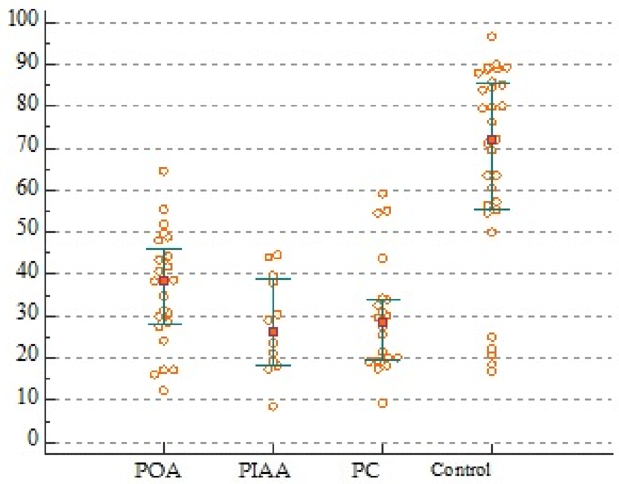

Figure S2 Distribution of the serum IL-8 level in patients with different origin of IAI on the 2nd - 3rd days of postsurgical intervention.

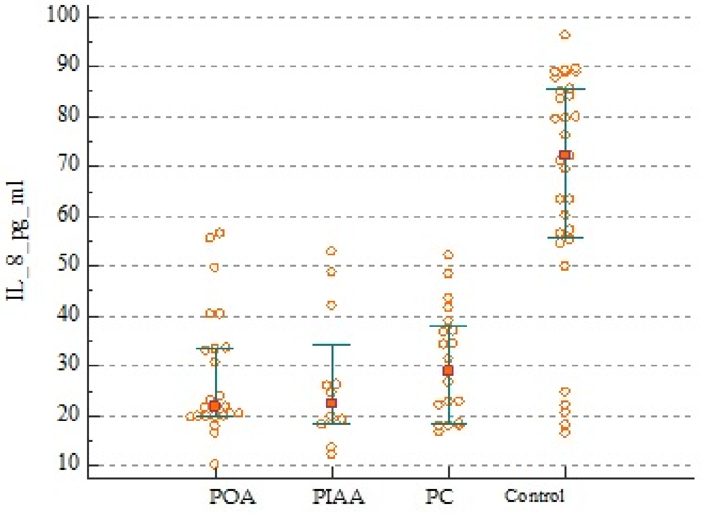

Figure S3 Distribution of the serum IL-8 level in patients with different origin of IAI on the 5th – 7th days of postsurgical intervention.

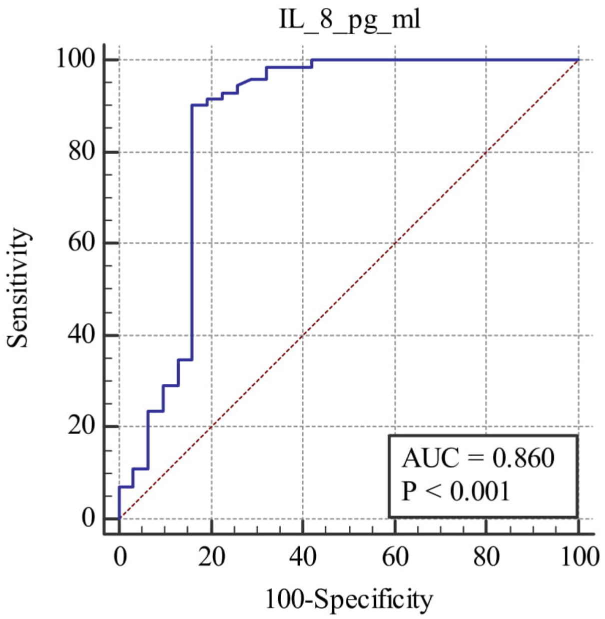

Figure S4. Results of ROC analysis for Postoperative Abscesses

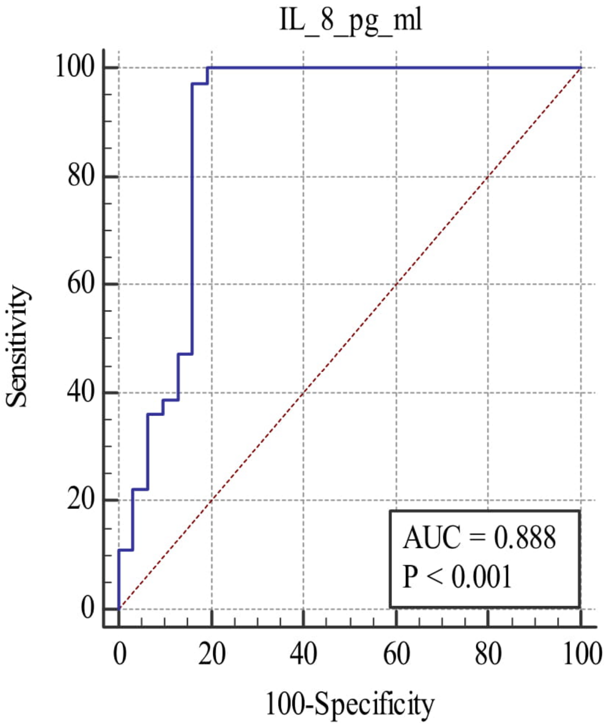

Figure S5. Results of ROC analysis for Postoperative Abscesses

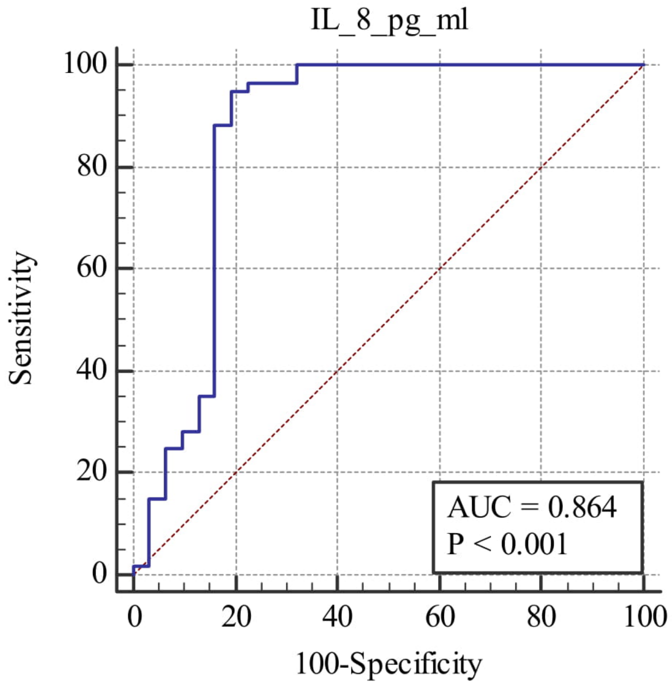

Figure S6. Results of ROC analysis for Primary Intra-Abdominal Abscesses

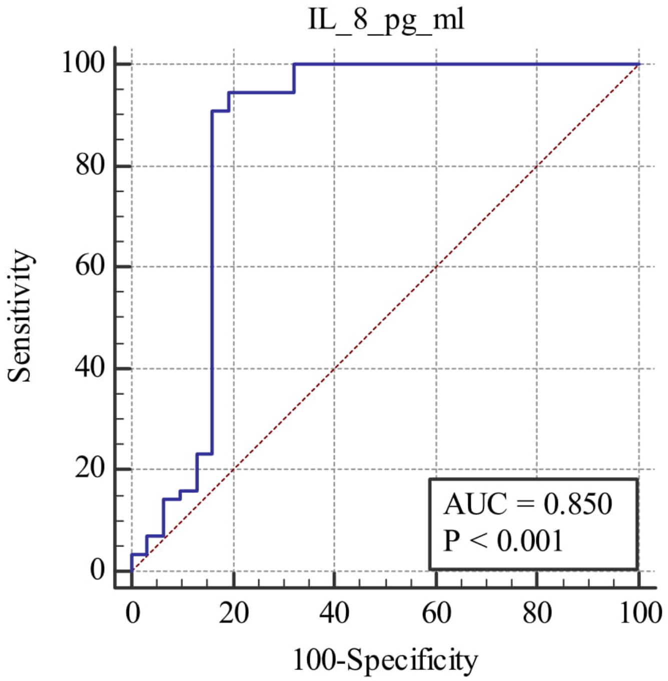

Figure S7. Results of ROC analysis for Peritoneal collection

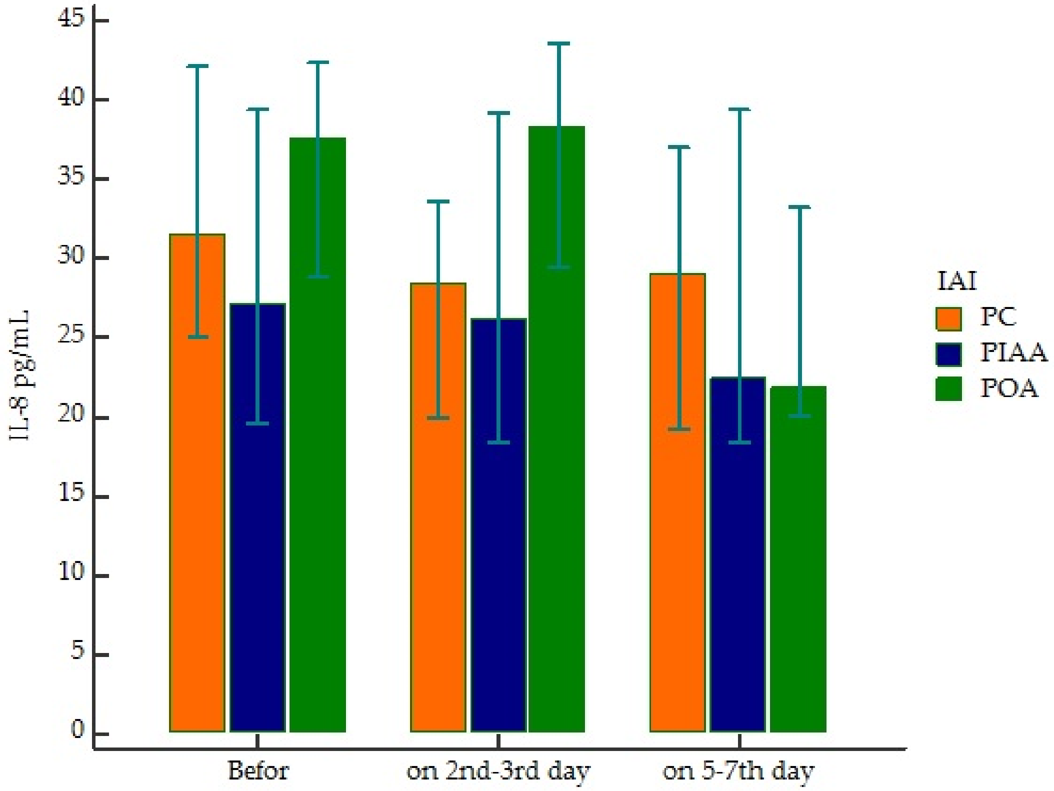

Figure S8. Result of two-way ANOVA test

Table S1 Two-way ANOVA: for serum IL-8 in patients with different origin of IAI

Dependent	IL_8_pg_mL
Sample size	168

Levene's test for equality of error variances

F	DF 1	DF 2	P
0,1916	8	159	0,992

Tests of Between-Subjects Effects

Source	Sum of Squares	DF	Mean Square	F	P
Time	638,242	2	319,121	2,075	0,129
Type_of_IAI	733,164	2	366,582	2,383	0,096
Time*Type_of_IAI	479,434	4	119,858	0,779	0,540
Residual	24458,110	159	153,825		

1. Time

Estimated Marginal Means

Time	n	Mean	Std. Error	95% Confidence interval
Befor	56	33,2967	1,7295	29,8810 to 36,7124
on 2nd-3rd day	56	31,3547	1,7295	27,9390 to 34,7703
on 5-7th day	56	28,3524	1,7295	24,9368 to 31,7681

Pairwise comparisons

Factors			Mean difference	Std. Error	P [a]	95% CI [a]
Befor	-	on 2nd-3rd day	1,9421	2,4458	1,0000	-3,9758 to 7,8599
	-	on 5-7th day	4,9443	2,4458	0,1347	-0,9736 to 10,8621
on 2nd-3rd day	-	Befor	-1,9421	2,4458	1,0000	-7,8599 to 3,9758
	-	on 5-7th day	3,0022	2,4458	0,6644	-2,9156 to 8,9201

on 5-7th day	-	Befor	-4,9443	2,4458	0,1347	-10,8621 to 0,9736
	-	on 2nd-3rd day	-3,0022	2,4458	0,6644	-8,9201 to 2,9156

[a] Bonferroni corrected

2. Type_of_IAI

Estimated Marginal Means

Type_of_IAI	n	Mean	Std. Error	95% Confidence interval
PC	60	31,5255	1,6012	28,3632 to 34,6878
PIAA	36	27,9769	2,0671	23,8944 to 32,0595
POA	72	33,5014	1,4617	30,6146 to 36,3882

Pairwise comparisons

Factors			Mean difference	Std. Error	P [a]	95% CI [a]
PC	-	PIAA	3,5486	2,6147	0,5300	-2,7779 to 9,8750
	-	POA	-1,9759	2,1680	1,0000	-7,2215 to 3,2697
PIAA	-	PC	-3,5486	2,6147	0,5300	-9,8750 to 2,7779
	-	POA	-5,5244	2,5317	0,0917	-11,6500 to 0,6011
POA	-	PC	1,9759	2,1680	1,0000	-3,2697 to 7,2215
	-	PIAA	5,5244	2,5317	0,0917	-0,6011 to 11,6500

[a] Bonferroni corrected

3. Time*Type_of_IAI

Estimated Marginal Means

Time	Type_of_IAI	n	Mean	Std. Error	95% Confidence interval
Befor	PC	20	34,4635	2,7733	28,9862 to 39,9408
	PIAA	12	29,2267	3,5803	22,1555 to 36,2978
	POA	24	36,2000	2,5317	31,2000 to 41,2000
on 2nd-3rd day	PC	20	30,0290	2,7733	24,5517 to 35,5063
	PIAA	12	27,7417	3,5803	20,6705 to 34,8128
	POA	24	36,2933	2,5317	31,2933 to 41,2934
on 5-7th day	PC	20	30,0840	2,7733	24,6067 to 35,5613
	PIAA	12	26,9625	3,5803	19,8914 to 34,0336
	POA	24	28,0108	2,5317	23,0108 to 33,0109

Summary statistics for dependent variable

Variable	Mean	Standard deviation
IL_8_pg_mL	31,6119	12,6129
